# Eukaryotic expression, Co-IP and MS identify BMPR-1B protein–protein interaction network

**DOI:** 10.1186/s40659-020-00290-7

**Published:** 2020-05-29

**Authors:** Jianlei Jia, Jipeng Jin, Qian Chen, Zan Yuan, Haiqin Li, Junhao Bian, Linsheng Gui

**Affiliations:** 1 Key of Laboratory of Plateau Ecology and Agriculture, Qinghai University, 251#, Ningda Road, Chengbei District, Xining, Qinghai 810016 China; 2 College of Agriculture and Animal Husbandry, Qinghai University, 251#, Ningda Road, Chengbei District, Xining, Qinghai 810016 China; 3 College of Animal Science and Technology, Gansu Agricultural University, Lanzhou Gansu, 730070 China

**Keywords:** Sheep, BMPR-1B, Co-immunoprecipitation, Mass spectrometry, Protein–protein interaction

## Abstract

**Background:**

BMPR-1B is part of the transforming growth factor β super family and plays a pivotal role in ewe litter size. Functional loss of exon-8 mutations in the BMPR-1B gene (namely the FecB gene) can increase both the ewe ovulation rate and litter size.

**Results:**

This study constructed a eukaryotic expression system, prepared a monoclonal antibody, and characterized BMPR-1B/FecB protein–protein interactions (PPIs). Using Co-immunoprecipitation coupled to mass spectrometry (Co-IP/MS), 23 proteins were identified that specifically interact with FecB in ovary extracts of ewes. Bioinformatics analysis of selected PPIs demonstrated that FecB associated with several other BMPs, primarily via signal transduction in the ovary. FecB and its associated interaction proteins enriched the reproduction process via BMP2 and BMP4 pathways. Signal transduction was identified via Smads proteins and TGF-beta signaling pathway by analyzing the biological processes and pathways. Moreover, other target proteins (GDF5, GDF9, RhoD, and HSP 10) that interact with FecB and that are related to ovulation and litter size in ewes were identified.

**Conclusions:**

In summary, this research identified a novel pathway and insight to explore the PPi network of BMPR-1B.

## Background

Ovarian folliculogenesis forms the base of ewe ovarian function, and is a key process in the production of mature oocytes during fertilization and hormone release during the estrous cycle [1]. Over the past decade, many studies had showed that ovarian folliculogenesis was regulated by members of TGF-β super family such as BMPs, inhibin, and activin [2]. BMPR-1B, a member of BMPs, is a major gene for the ovine ovulation rate, and plays a pivotal role in follicle development and litter size [3]. Previous studies identified that the A746G mutation, a loss-of-function mutation in the BMPR-1B gene, generates a protein mutant that promotes steroid production and ovulation rate. Consequently, this mutation increased litter size in Australian Merino sheep, and was named the FecB gene (or the FecB mutation of BMPR1B gene) [4]. The FecB gene was also identified as a major gene for litter size in Small Tail Han sheep and Hu sheep [5, 6]. Bone morphogenetic protein receptor 1B contains three parts: An N-terminal extracellular ligand binding domain, a single transmembrane region, and a C-terminal serine/threonine kinase domain [7]. Compared with BMPR-1B, the mutant protein results in the substitution of the 249th amino acid from glutamine to arginine (Q249R). Consequently, upon binding with BMP ligands, BMPR-IIB transphosphorylates the GS domain of the BMPR-1B, which leads to the activation of downstream cascades and the inactivation of the partial receptor [8]. As a result of this, the inhibiting effect of steroidogenesis on GDF5 and BMP4 is weakened, the SMAD state of expression and phosphorylation is changed, the synthesis of estradiol by FSH induction is promoted, the synthesis and secretion of progesterone is inhibited, granular cell differentiation of mutation ewes and follicular maturation are accelerated, and ovulation is increased [9]. Although little is known about the follicular regulatory mechanism of BMPR-1B in single, twins, and multiple prolific ewes, these properties of BMPR-1B can provide new concepts for studies of ewe follicular development and litter size.

It has been reported that BMPR-1B exerts additive effects on the ovulation rate, which could be increased by 1.5 to 2.0 for each copy in Booroola Merino sheep [7]. Furthermore, BMPR-1B inhibited granular cells apoptosis, prevented follicular atresia, and promoted ovulation and litter size. This may be an important physiological mechanism with which BMPR1B affects fecundity in sheep [8]. Miao compared the ovarian proteomes of Han ++ and Han BB ewes have indicated that Han BB ewes higher ovulation rate may relate to mitochondrial oxidation functions protein expressions, and it could provide a prospective understanding of the molecular mechanism for high prolificacy of Small Tail Han sheep [10]. The development of post-genomics, proteomics, transcriptomics, metabolomics, and bioinformatics offers unprecedented advantages for research on the biological function of genes and underlying biological molecular regulatory mechanisms. This study applied eukaryotic expression, Co-IP, and MS to BMPR-1B, a signal transduction protein found in intracellular and extracellular signal transduction pathway in Smads, p38-MAPK, and Erk-MAP.

To explore the biological functions of BMPR-1B and its resulting protein in ewe folliculogenesis, a eukaryotic expression vector of pcDNA3.1a-BMPR-1B was constructed for the highly efficient expression of BMPR-1B protein in SF9. Furthermore, a monoclonal antibody was prepared and a CoIP-MS approach was used to identify BMPR-1B protein–protein interactions (PPIs). In addition, the signal pathway of target proteins was analyzed and bioinformatics prediction indicated that BMPR-1B interacted with proteins that promoted ovulation in ewe ovary. CoIP-MS identified 23 BMPR-1B proteins with significant correlation, including PPIs with many Smads. These results associated BMPR-1B with reproduction processes and signal pathways. The target proteins were primarily related to developmental processes, multicellular organismal processes, and biological regulation. The result also showed that BMPR-1B interacted with several binding proteins, such as BMP2, BMP4, GDF5, GDF9, RhoD, and HSP 10. The Co-IP/MS results were further assessed by western blotting. In summary, these results indicated a new concept of BMPR-1B PPIs and their biological functions for sheep litter size. Furthermore, this study laid a technical foundation for the in vitro expression of sheep BMPR-1B. For the further study of the biological function and specificity of developed diagnostic reagents, BMPR-1B was identified the major gene for sheep.

## Materials and methods

Experimental ewes with clear lambing record were killed by bloodletting via the carotid artery. The ovaries of Small Tailed Han sheep were collected from a slaughter house in accordance with approved guidelines, and ovary samples were peeled and washed to remove all surface fat and ligaments. The ovaries were then transferred to sterile plastic tubes and stored at − 80 °C.

### Detection of FecB mutations

A primer pair was designed to detect single nucleotide polymorphisms in exon8 of the BMPR-1B gene in blood samples of ewes. The procedure used PCR-RFLP and followed the description of Wang [11]. The primer sequences were as follows: F: 5′-GTCGCTATGGGGAAGTTTGGATG-3′, R: 5′-CAAGATGTTTTCATGCCTCATCAACACGGTC-3′.

### Gene cloning

BB and ++ genotype ewes were selected, and the total RNA of their ovaries was extracted using an RNA extraction kit (TaKaRa, China). After the RNA was reverse transcribed to cDNA, it was amplified in a volume of 25 µl. Amplification was performed for 35 cycles using DNA thermal cycler (Bio-Rad, USA). The first cycle was at 94 °C for 2 min followed by 35 subsequent cycles of 94 °C for 30 s, 59.5 °C for 30 s, then 72 °C for 60 s, and the last cycle at 72 °C for 7 min.

The PCR products were isolated from the agarose gel with a MiniBEST Agarose Gel DNA Extraction Kit (TaKaRa, China). 5 μL of purified PCR products (BMPR-1B/FecB) were ligated into 0.5 μL of pMD19-T with 5.5 μL Solution I using T4 DNA ligase at 4 °C overnight. 10 μL of transformed ligation mixture was inserted into 100 μL of *Escherichia coli* DH5a in LB agar containing X-gal, IPTG, and Ampicillin. This ligation mixture was transformed into *E. coli* DH5a using an ice-water bath for 30 min and heat shock at 42 °C for 45 s, followed by incubation on ice for 1 min. The transformation products were cultured on LB agar overnight. Positive clones were sequenced by Sangon Biotech (China).

### Eukaryotic expression recombined target gene

pcDNA3.1a (eukaryotic expression plasmid vector) and pMD19-T-BMPR-1B/FecB (cloning vector) were digested with BamH I and Xho I (TaKaRa, China) at 37 °C for 4 h. The digested products were identified by 2% agarose gel electrophoresis. The empty vector and objective gene fragment were recovered from agarose, using a gel extraction kit (TaKaRa, China). The empty vector was ligated into the objective gene fragment using T4 DNA ligase (TaKaRa, China) at 16 °C overnight. Ligation products were cultured in LB agar containing ampicillin at 37° C overnight. Positive clones were sequenced by Sangon Biotech (China).

Protein expression of each gene was assessed using a 10 ml suspension culture of *Spodoptera frugiperda* (SF9) cells (Invitrogen, USA). In a 2.0 ml tube, 25 μL of plasmid DNA (25 μg) was diluted in 10% fetal calf serum Grace’s medium (Invitrogen, USA). 150 μL of cell transfection reagent (QIAGEN, Germany) was diluted in 1.5 ml of the same medium in a 2.0 ml tube. The diluted plasmid DNA was then added dropwise to the diluted cell transfection reagent and the mixture was gently blended to avoid precipitation, and then left at room temperature for 20 min. Finally, the entire mixture was added to the cells and incubated at 70 rpm on a shaking table at 28 °C until the cytopathic effect (CPE) exceeded 80% (72–96 h. The transformed cells and total cells were counted with inverted microscope, respectively. 5–7 microscopic fields were randomly selected in 400 × lens, and more than 700 total cells were counted. The average transfection rate: Transfection rate = the number of transformed cells/total cells × 100%). Then, supernatants and precipitation were separately collected, and proteins were separated by SDS-PAGE. The recombinant protein was purified using the Ni–NTA Superflow Protein Purification Kit (QIAGEN, Germany).

### Generation of anti-BMPR-1B and anti-FecB monoclonal antibodies

MAbs production was conducted by GenScript (USA). BAL b/c mice were immunized with the above-described purified BMPR-1B/FecB proteins and the production of specific hybridomas was performed following standard protocols. Clones 3M2/R1, 2D8/F1, and 1G6/H4 were selected as hybridomas for this study. They were produced anti-BMPR-1B antibodies, clones 8G2/T3, 4F7/G6, and 1M0/H10, which produced anti-FecB antibodies. 3A10/B8 that produced control antibodies.

### Identification of the BMPR-1B/FecB interacting proteins in the ovary of ewes

Each Co-immunoprecipitation (Co-IP) was conducted three times. For Co-IP, the BMPR-1B/FecB and interacting proteins in ewes’ ovary were enriched using the Mag sProtein A/G Co-Immunoprecipitation Kit (BioCanal, China). For regular Co-IP, 0.1 g extracts from the ovaries of ewes were lysed with 1 ml lysis buffer at 4 °C for 30 min and sonicated in a water bath sonicator at amplitudes of 22% for 60 s (0.1 s on and 1 s off) [12]. 5 mg of total protein was used for immunoprecipitation with 100 μg of IgA.

The BMPR-B/FecB and interacting proteins were co-immunoprecipitated from whole ewe ovary extracts via Mag sProtein A/G Immunoprecipitation Kit and regular Co-IP. The empty vector, which was expressed by SF9 cells, served as negative control. The immunoblot results indicated that the interacting proteins of BMPR-1B/FecB were enriched in the pulldown. In contrast, BMPR-B/FecB and interacting proteins were not identified in the control vector pulldown. This demonstrated that the method was efficient, specifically co-immunoprecipitated BMPR-B/FecB, and interacted with proteins from the ovary extracts of ewes.

### Protein identification and bioinformatics analyses

LC–MS analysis was conducted by Applied Protein Technology (Shanghai, China). 5800 MALDI-TOF/TOF (AB Sciex, USA) was used for mass spectrometer (MS) data analyses. For quantitative analysis, a protein must have at minimum one unique peptide match with the MS ratios. A ≥ 3.0 or ≤ 3.0-fold cutoff value was used to identify up-regulated and down-regulated proteins with a *p* value < 0.05.

Functional annotation and classification of all identified proteins were determined by using the Blast2GO program against the uniprot database. Pathway analyses were extracted using the search pathway tool of the KEGG mapper platform (http://www.genome.jp/kegg/mapper.html). Pathway enrichment statistics were conducted by Fisher’s exact test, and the pathways with a corrected p value < 0.05 were defined as the most significant pathways. The search tool for the retrieval of interacting genes/proteins database for the prediction of the physical and functional interactions was used to analyze the PPI network. The graphical visualization and analysis of interaction network was performed in GeneMANIA software.

## Results

### Detection of polymorphism in the BMPR-1B (FecB) gene

**Fig. 1:**
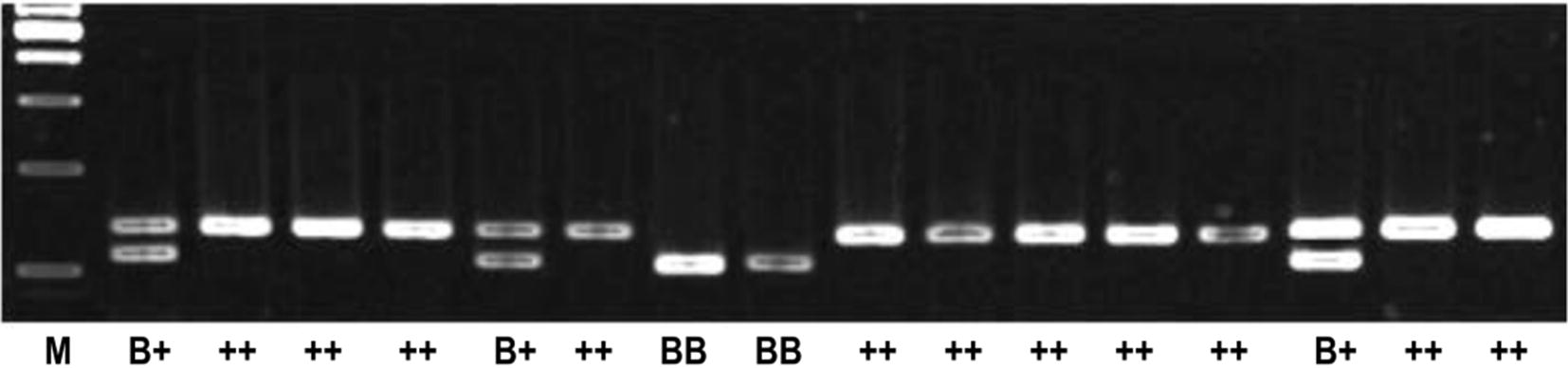
The FecB mutation of BMPR-1B gene was digested by AvaII

DNA was extracted from the whole blood of ewes using a DNA preparation kit (TaKaRa, China). The concentration and purity of the extracted genomic DNA were determined by spectrophotometer (Germany). The PCR product was about 140 bp, located on exon 8 of the BMPR-1B gene. The FecB mutations could be identified via RFLP method using Restriction Endonuclease Ava II. The PCR product of exon 8 of the BMPR-1B gene can generate two alleles by Ava II digestion: the wild-type allele (+) and the mutant allele (B). The wild-type allele (+) was not sensitive to Ava II and could not be cut, while the mutant allele (B) was cut by Ava II, which yielded two DNA fragments of 110 and 30 bp. Analysis showed that the A746G of BMPR-1B gene could sensitize it to Ava II, and the sequence (G|GACC) of FecB mutation was recognized as the site of cutting (Fig. 1).

### Eukaryotic expression recombined gene BMPR-1B and FecB

**Fig. 2:**
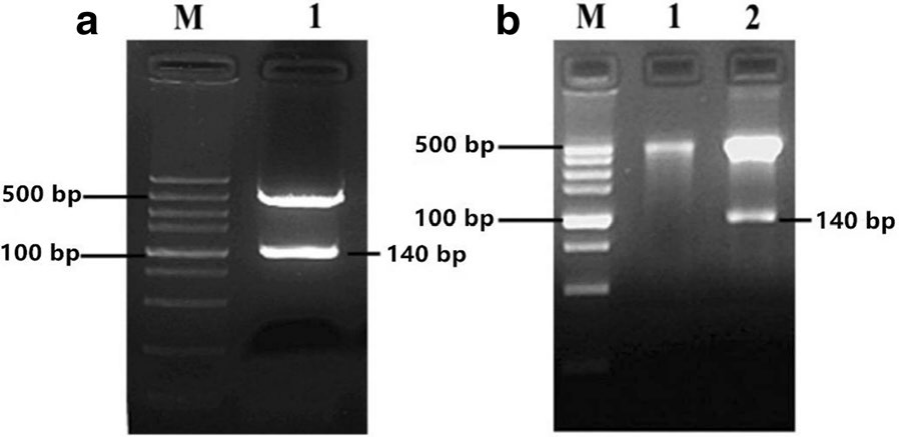
Agarose electrophoresis of isolated plasmid at native condition (**a**) and at digested with BamH I and Xho I enzymes (**b**). **a** M was DNA marker, 1 was recombinant plasmid. **b** 1 was empty plasmid, 2 was recombinant plasmid

Agarose electrophoresis identified two bands in the pMD19-T-BMPR-1B/FecB plasmid and only one band in the pMD19-T-NP plasmid. Figure 2 confirmed that there was a 140 bp band (DNA fragment) by cleavage of pcDNA3.1a-BMPR-1B/FecB plasmid by plasmid digestion of BamH I and Xho I restriction endonuclease. However, for the pMD19-T-NP, the target 140 bp band (DNA fragment) did not appear.

### Generation of anti BMPR-1B and anti FecB monoclonal antibodies

**Fig. 3:**
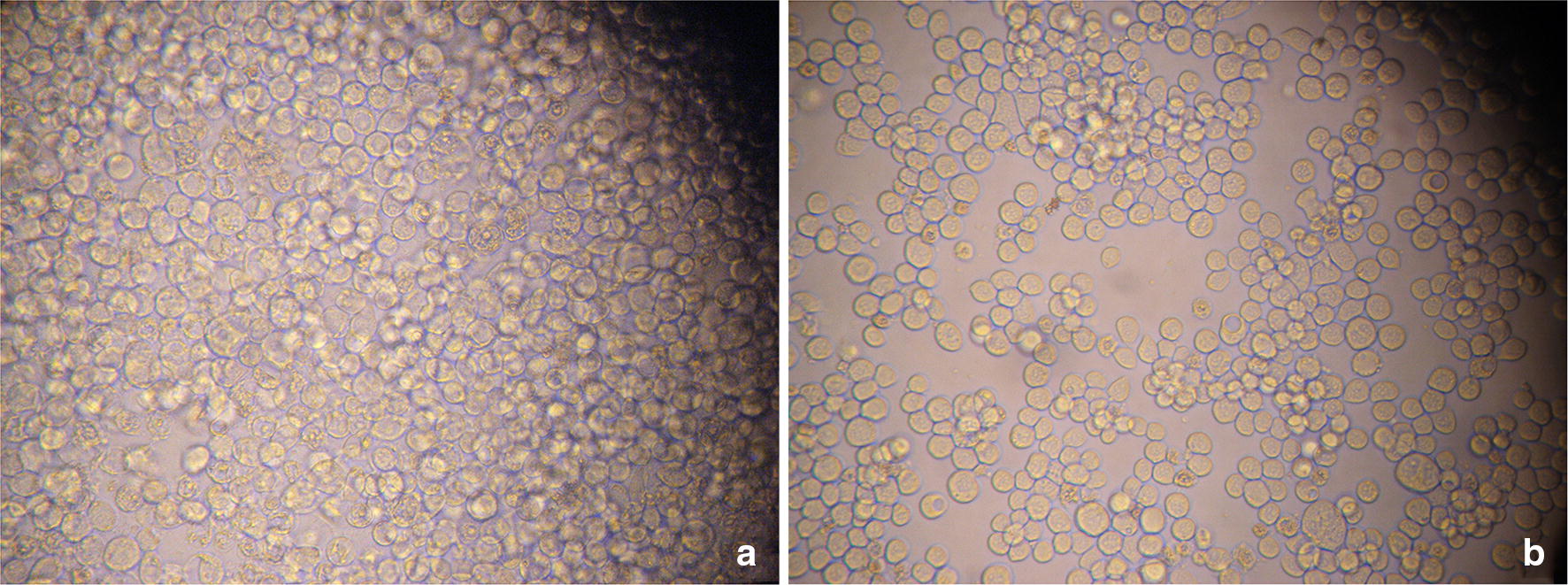
Sf9 of post-transfection (**a**) and Sf9 of Normal (**b**)

After transfection, SF9 cells were monitored for CPE by inverted microscope. And the pcDNA3.1a-BMPR-1B/FecB transfected group showed the CPE of visible to naked eye compared with controls 24 h later (Fig. 3). After 7 days of monitoring, the result indicated a CPE expression of 80% between 72 and 96 h post-transfection.

**Fig. 4:**
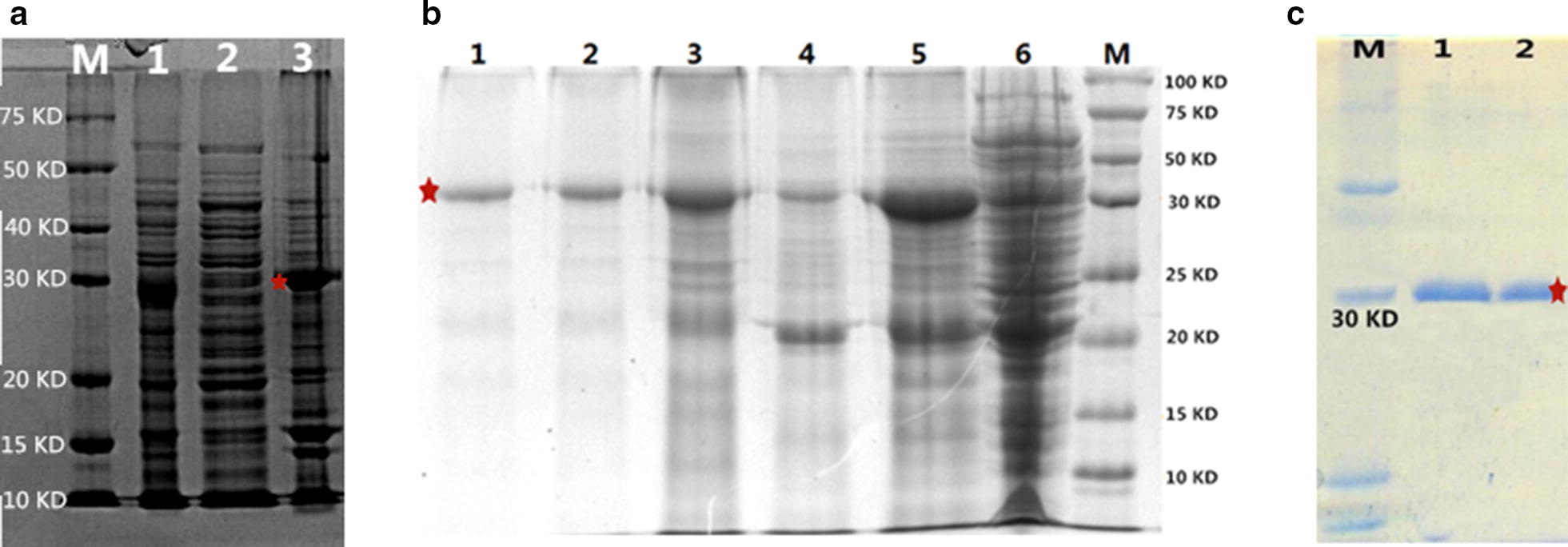
**a** SDS-PAGE of Protein extraction M was Marker; 1 was SDS-PAGE of total protein; 2 was SDS-PAGE of supernatant; 3 was SDS-PAGE of precipitation. b Purification of BMPR-1B recombined protein M. Protein marker; 1–3. Purified recombined protein; 4. Flow through; 5. Unpurified inclusion bodies; 6. The total protein before induction. **c** Western blotting of antibody

The supernatants and precipitate were analyzed by SDS-PAGE in an 8% polyacrylamide gel, and coomassie brilliant blue R-250 staining was used to visualize target proteins. The recombinant proteins were mainly identified via precipitation (Fig. 4a), and the Ni–NTA Superflow Protein Purification Kit (Fig. 4b) was used to purify recombinant proteins. Figure 4c shows that the specific hybridomas and monoclonal antibody were produced by standard protocols via BAL b/c rats, using the purified proteins described above. The ELISA method was used to detect the BMPR-1B/FecB antibody titer, and the serum antibody titer was measured when positive/negative (P/N) exceeded or was equal to 2.1 on OD 450 nm. The results showed that the high-titer rat anti-BMPR-1B/FecB serum had been prepared by four immunizations. Then, the monoclonal antibodies were typed by ClonaCell Kit (StemCell, Canada).

### Identification of the BMPR-1B interacting proteins in ovary extracts of ewes

**Fig. 5:**
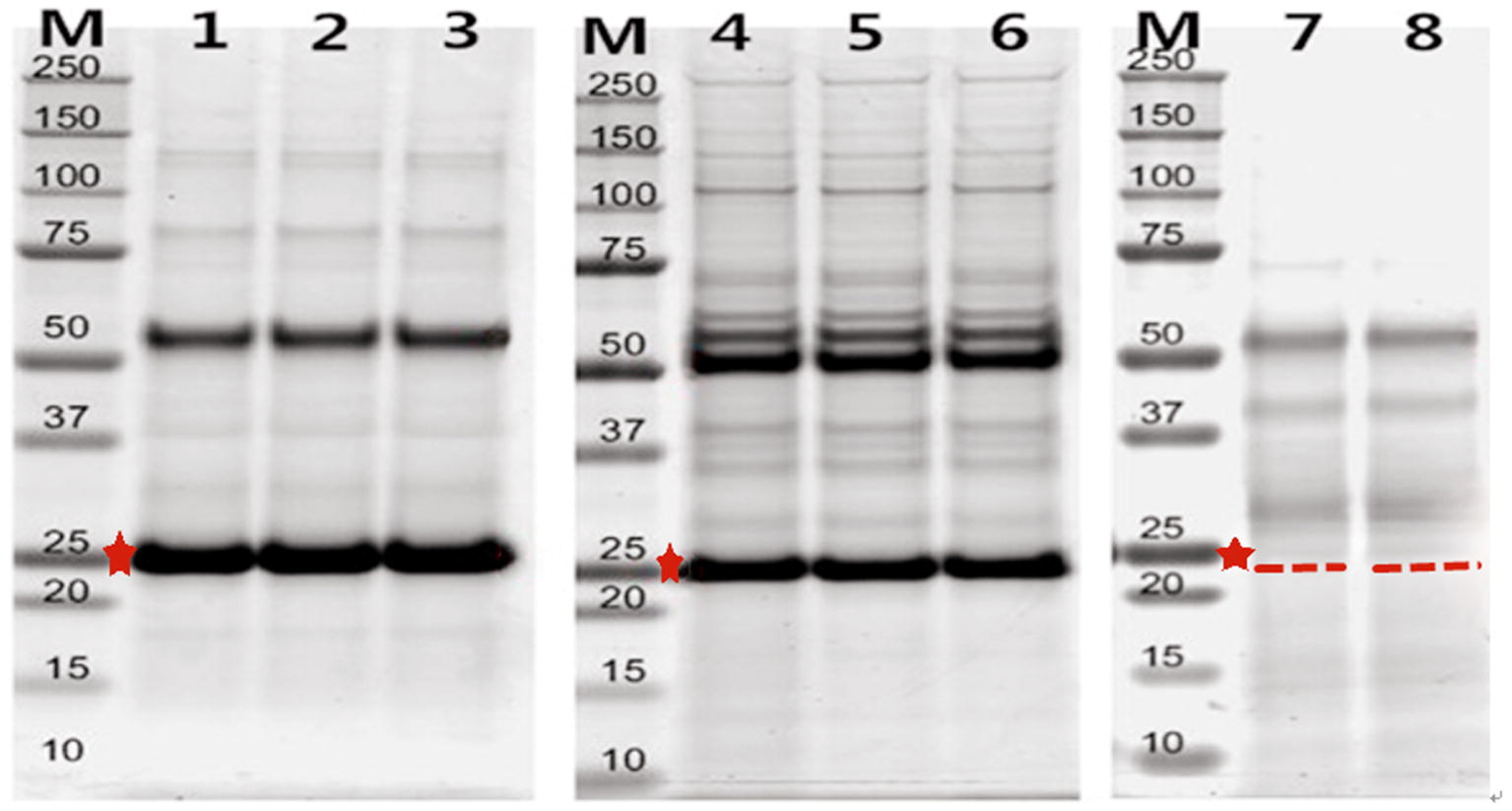
Identification of BMPR-1B/FecB interacting proteins

The BMPR-1B/FecB interacting proteins were efficiently and specifically screened from ovary extracts of ewes via SDS-PAGE (Fig. 5). Coomassie staining gels showed that several bands only appeared in Co-IP of BMPR-1B (samples 1, 2, and 3) and Co-IP of FecB (samples 4, 5, and 6), but were not present in the control vector (samples 7 and 8).

Sixty-three immunoprecipitated proteins were successfully identified with *Ovis aries* or *Bos taurus* database matching in Mascot. Then, the most likely associations between these target proteins and BMPR-1B/FecB were computed by the manual thresholding approach and a probabilistic PPI prediction algorithm, which identified 34 high-confidence candidates. Table 1 is our 24 of these 34 proteins were at least threefold more abundant between CoIP of BMPR-1B and FecB groups. BMPR-1B protein and 23 target CoIP proteins, and Table 1 is in identification of the BMPR-1B interacting proteins in ovary extracts of ewes part of paper result.

**Table 1:** Merged list of identified BMPR-1B interacting proteins from CoIP and MS

Sample	Protein name	No. NCBI	*Data sources*	Molecular weight	PI
1	BMPR-1B	gi|57164243	*Ovis aries*	32018.6	6.67
2	SOD[Cu–Zn]-like	gi|426237454	*Ovis aries*	15856	6.14
3	Transgelin	gi|426244582	*Ovis aries*	25598.9	9.06
4	MTHFR	gi|281833111	*Bos taurus*	15517.9	3.9
5	Histone H3	gi|426250704	*Ovis aries*	15536.5	11.24
6	GDF9	gi|402244329	*Ovis aries*	19644.6	4.76
7	Calmodulin	gi|262073073	*Bos taurus*	16703.3	4.1
8	Aldose reductase	gi|426228047	*Ovis aries*	36291.6	5.95
9	Hemoglobin subunit mu	gi|139947644	*Bos taurus*	16120.3	6.75
10	Alpha globin chain	gi|1789	*Ovis aries*	15283.9	7.94
11	MRCL3	gi|223633898	*Ovis aries*	19851.5	4.72
12	Myosin light chain 6	gi|165875535	*Ovis aries*	16094.8	4.6
13	Galectin-1	gi|47779226	*Ovis aries*	15150.4	5.02
14	Annexin A2	gi|147899370	*Ovis aries*	38872.9	6.92
15	Glyceraldehyde-3-phosphate dehydrogenase	gi|296785215	*Ovis aries*	36110.4	8.51
16	Glutathione S-transferase Mu	gi|426216178	*Ovis aries*	21331.9	8.42
17	PEBP	gi|426224189	*Ovis aries*	21082.7	6.51
18	Nitric oxide-inducible gene protein	gi|426252227	*Ovis aries*	112526	7.03
19	Beta-A globin chain	gi|86129749	*Ovis aries*	16050.3	6.49
20	BMP2	gi|353681895	*Ovis aries*	11258.92	9.96
21	BMP4	gi|160333315	*Ovis aries*	46611.90	8.57
22	GDF5	gi|196119889	*Ovis aries*	7456.80	10.01
23	RhoD	gi|803191482	*Bos taurus*	21927.32	8.76
24	HSP 10	gi|27805927	*Bos taurus*	10931.69	8.89

### Validation of selected BMPR-1B/FecB PPIs

**Fig. 6:**
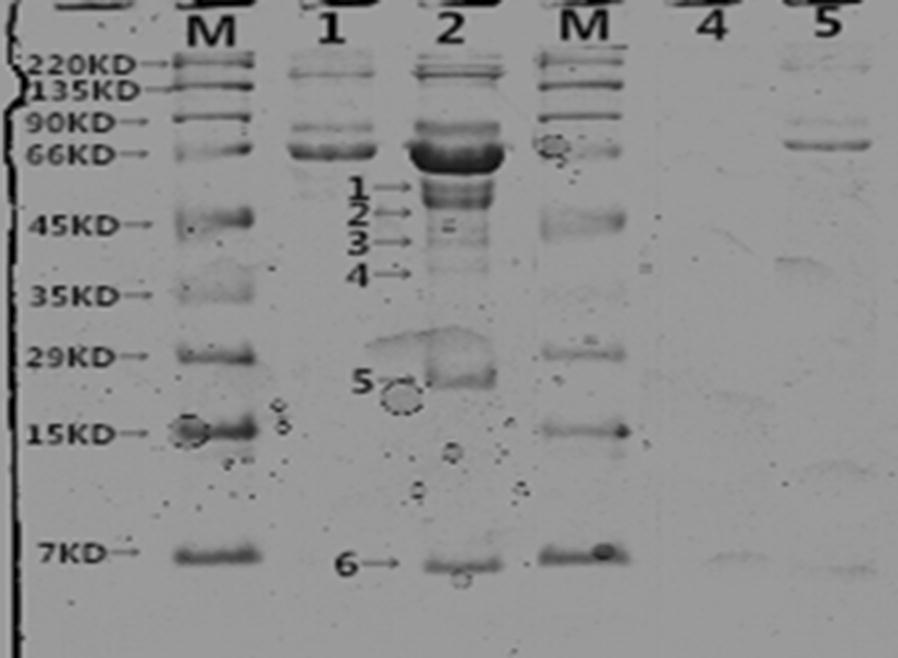
SDS-PAGE of BMPR-1B interacting proteins of ewes’ ovary extracts by CoIP Lanes: M was Marker; 1 was control group; 2 was experimental group; 3 was supernatant of antibody for CoIP; 4 was NP control; 5 was supernatant of antigen for CoIP. Bands: 1 was BMP4; 2 was RhoD; 3 was GDF9; 4 was BMP2; 5 was HSP10; 6 was DGF5

**Fig. 7:**
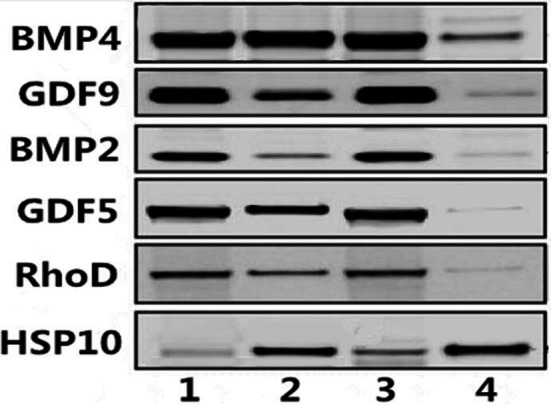
Western blotting of BMPR-1B/FecB interacting proteins. The pulldown of interacting proteins were immunobloted with BMPR-1B/FecB antibodies. The same pulldown were also immunobloted with BMP 4, GDF9, BMP2, GDF5, RhoD and HSP10 antibodies. 1 was verification of BMPR-1B antibodies, 2 was verification of FecB antibodies, 3 was verification of target interacting proteins antibodies

Previously research showed that as the natural ligand of BMPR-1B, GDF5 and BMP4 participated in the ewe ovarian folliculogenesis. Both proteins were detected in the current Co-IP/MS study. Furthermore, specific interactions of several identified candidate proteins with BMPR-1B/FecB were identified by CoIP, followed by immunoblot: BMP2, GDF9, RhoD, and HSP 10 (Fig. 6). All these candidate proteins were identified by Western blotting (positive and negative validation, see Fig. 7). In summary, the PPIs prediction results confirmed the specific interactions of target proteins with BMPR-1B/FecB, further providing evidence of the validity for the Co-IP/MS approach.

### Gene ontology and pathway analysis

**Fig. 8:**
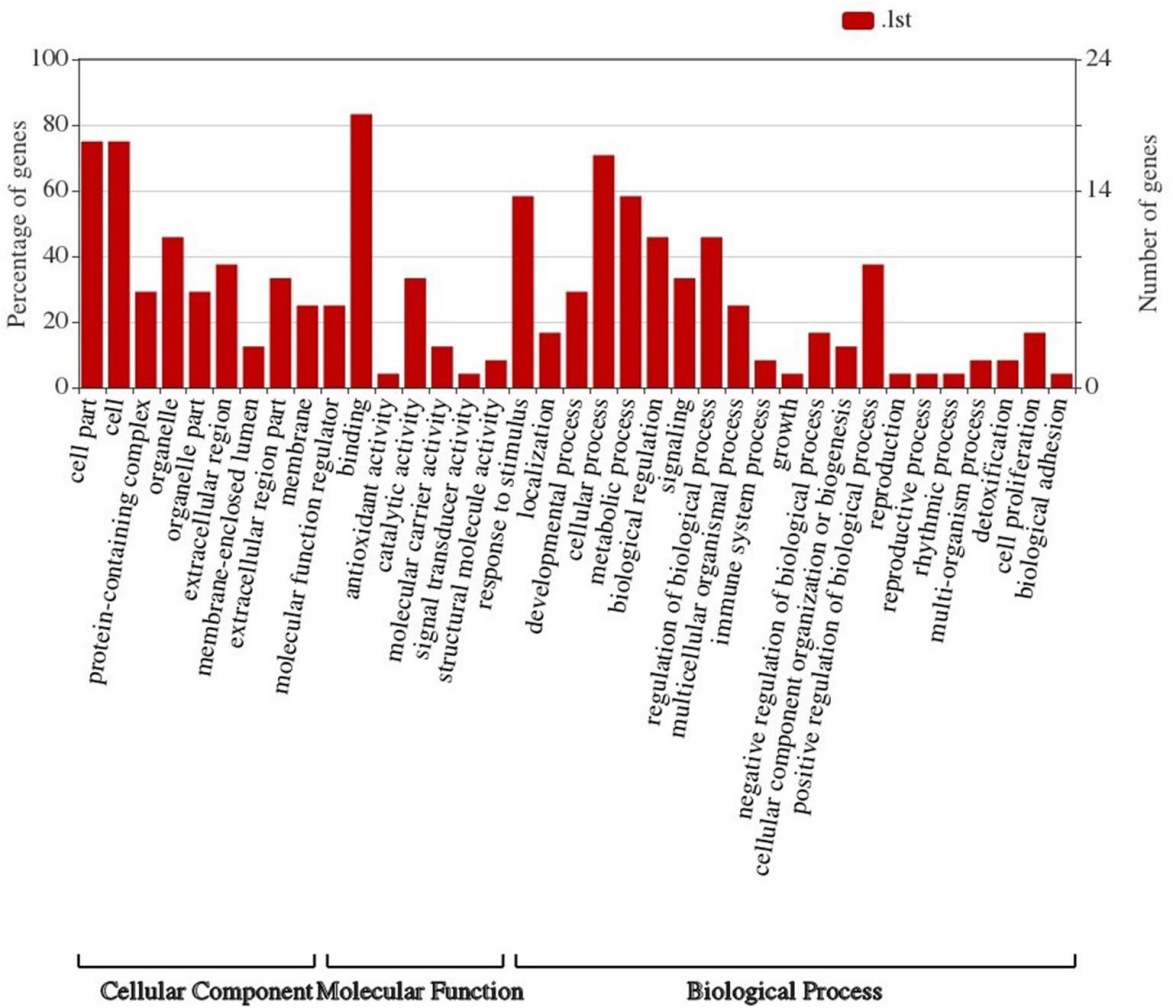
FecB-interacting proteins level 2 GO terms

To explore biological processes associated with FecB-interacting proteins, enrichment analysis in the Gene Ontology (GO) domain “Biological Process” was performed (Fig. 8). The results identified two predominant themes: binding and signaling. For the signaling term, the pivotal term in ewe ovarian folliculogenesis mainly related to signal transduction (e.g., “TGF-beta signaling pathway” and “Cytokine-cytokine receptor interaction”). Other developmental process terms included “Biological regulation”, “Cellular process”, “Growth”, and “Metabolic process”. The terms unrelated to these phenomena and unconnected to any nodes included “Detoxification” and “Immune system process”.

**Fig. 9:**
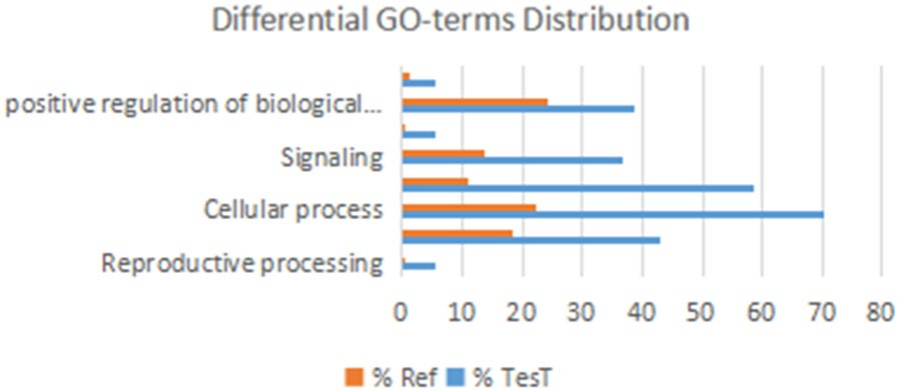
Enrichment GO-terms Analysis

To provide further insight into the biological processes identified by this approach, Fishers’ test (significance A/B Test) was applied to target terms (Fig. 9). Terms with the highest number of associated proteins from these results were the proteins that resulted in the identification of an enriched biological process. For example, the identification of the “Signaling” and “Reproduction” terms were in large part the result of the large number of TGF-β family proteins in the list of BMPR-1B-interacting proteins. In contrast, the enrichment for “Regulation of translation” resulted from the presence of initiation and elongation factors (e.g., BMP2 and BMP4) in the list of BMPR-1B/FecB-interacting proteins.

**Fig. 10:**
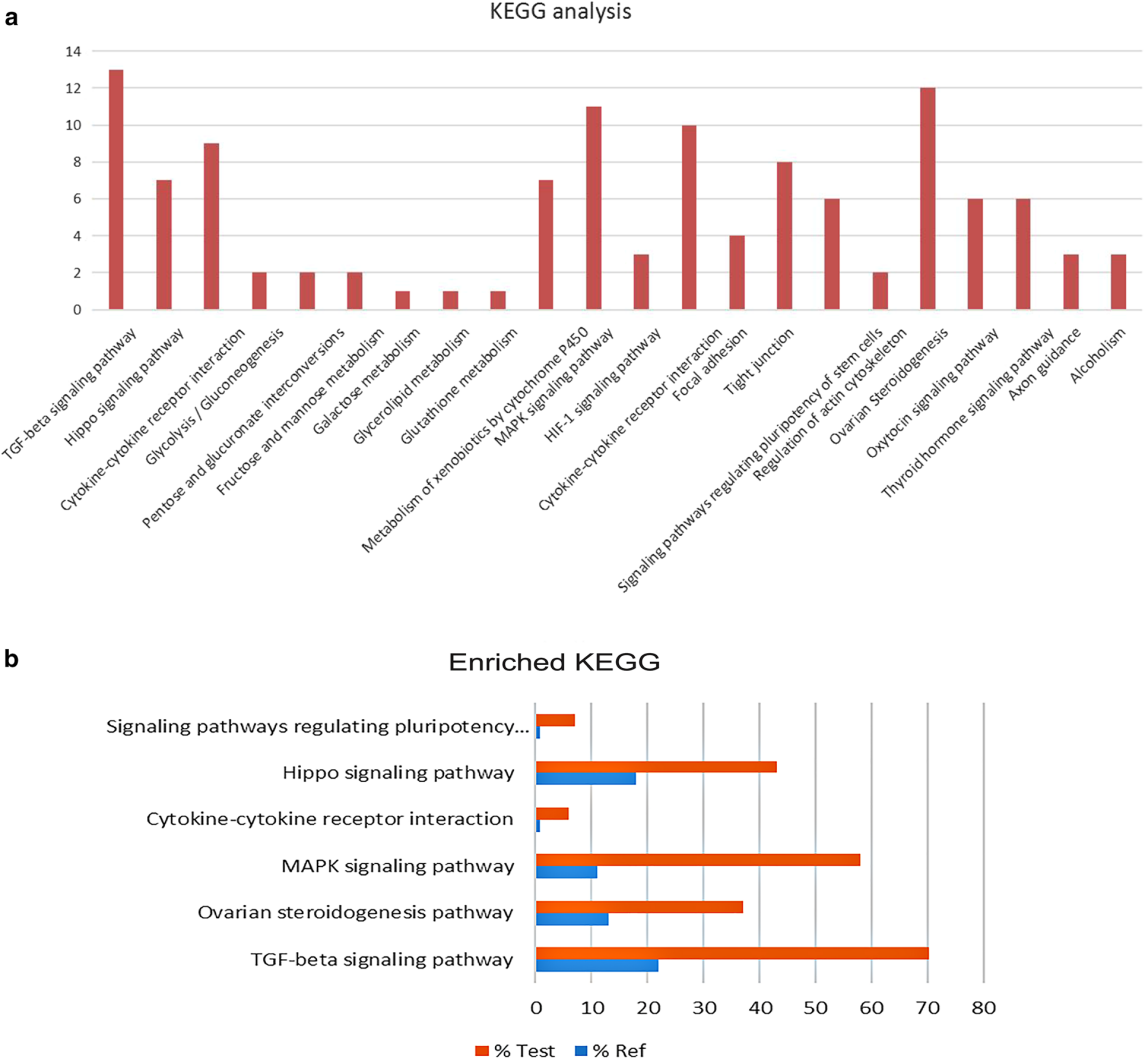
**a** FecB-interacting proteins KEGG pathway. b Enrichment KEGG pathways analysis. Fisher’s Exact Test, P value < 0.01

The major pathways associated with FecB-interacting proteins were identified using KEGG Pathway Analysis via KAAS software. Twenty-eight of total of 103 pathways were significantly enriched (p < 0.05). The significantly enriched pathways were “TGF-beta signaling pathway”, “Ovarian steroidogenesis pathway”, “MAPK signaling pathway”, “Cytokine-cytokine receptor interaction”, “Hippo signaling pathway”, and “Signaling pathways regulating pluripotency of stem cells” (Fig. 10a, b). The results of KEGG pathway analysis matched the results of gene ontology analysis.

### Protein–Protein interaction pathway prediction analyses

**Fig. 11:**
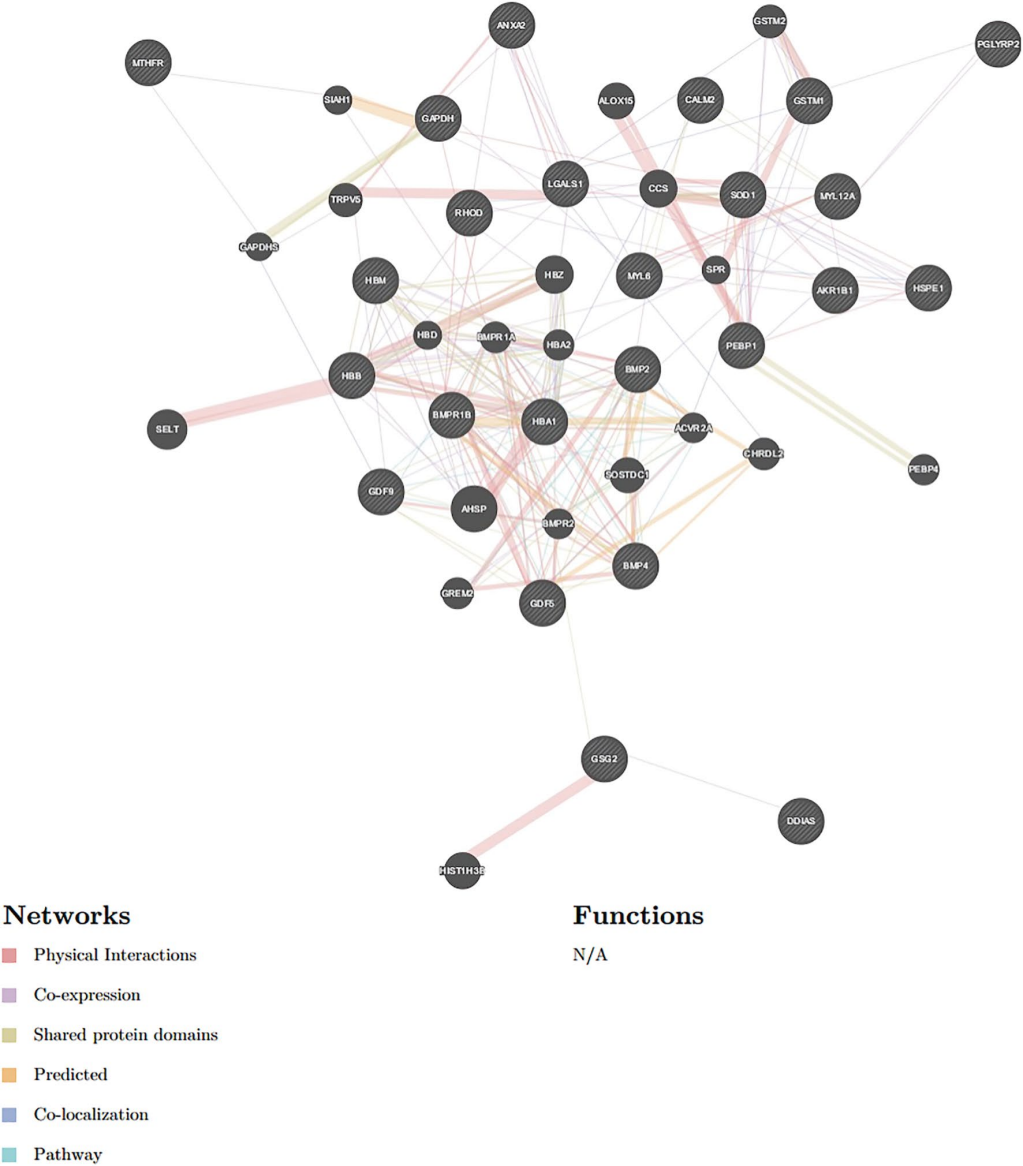
Protein-protein interaction networks of BMPR-1B/FecB in ewe’s ovary extracts based on GeneMANIA software

**Fig. 12:**
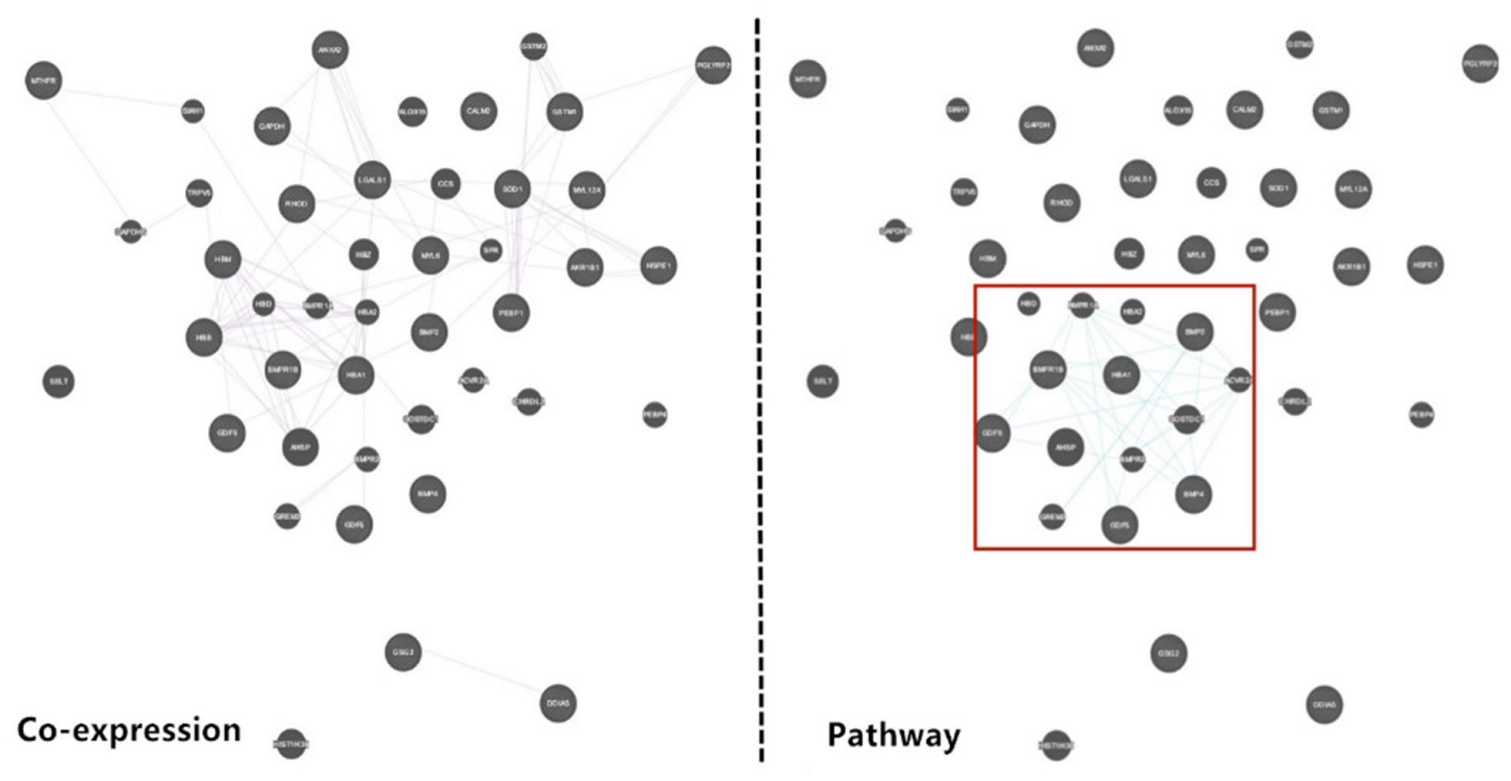
Protein-protein interaction networks of BMPR-1B in ewe’s ovary extracts based on GeneMANIA software of Co-expression and pathway

**Fig. 13:**
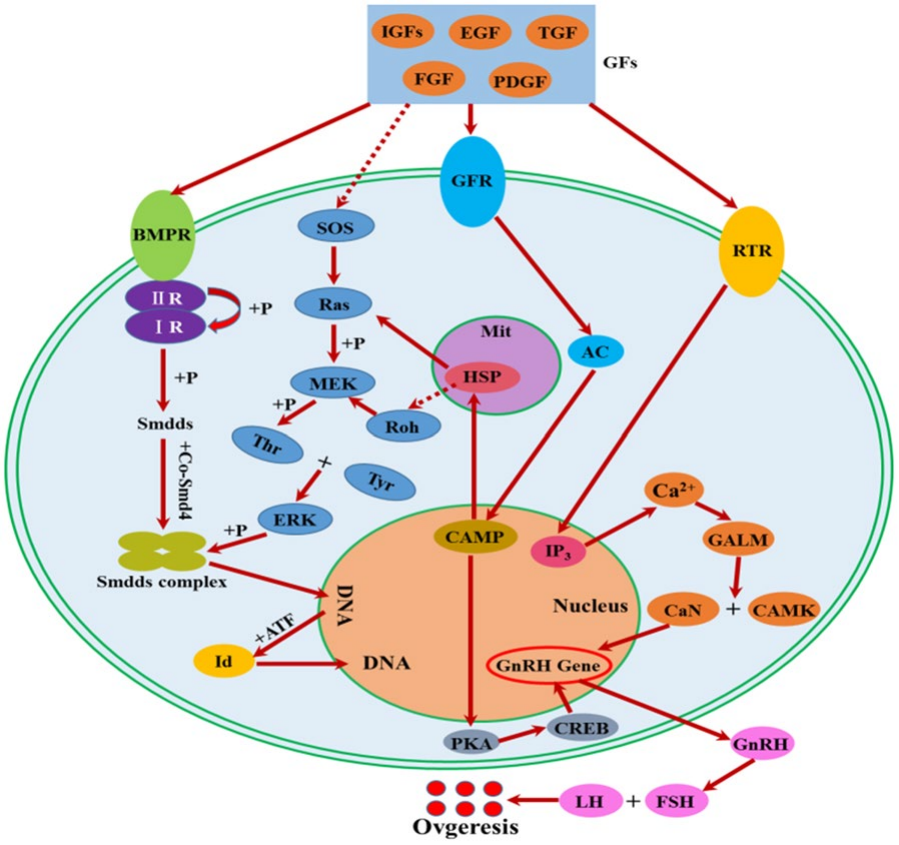
BMPR-1B-interacting proteins of sheep oocyte development and ovulation

To explore the information of the FecB-interaction network, connected proteins among the 24 proteins were analyzed via GeneMANIA software and Uniport database. As reported, BMPR-1B, BMP2, BMP4, GDF5, GDF9, and HSP10 constituted a complex and strong PPI network (Fig. 11). Moreover, interestingly, the target proteins displayed different mutual interections with other pathways in the complex PPI network between co-expression and pathway (Fig. 12). Therefore, based on these experimental results, an BMPR-1B/FecB pathway that could regulate sheep oocyte development and ovulation (Fig. 13) was designed. The predicted pathway corroborated the importance of pathways related to signal transduction for ewe oocyte development and ovulation.

## Discussion

Gonadotropins, such as follicle stimulating hormone (FSH) and luteinizing hormone (LH), are usually considered to regulate ewe ovarian oocytes maturity and ovulation. Research confirmed that various intra-ovarian factors, including BMPR-1B, could control the release of these gonadotropin hormones (FSH and LH) [13]. A recent study showed that the BMPR-1B/FecB gene was widely expressed in ovary tissues such as granulocytes, oocytes, follicles, and corpus luteum [14]. Miao et al. explored the mechanisms for sheep fecundity on protein level, they found FecB mutation ewes had a lower expression level in ribosome related protein, Bioinformatics analysis showed that Oxidative phosphorylation occurred in 10.07% different expression proteions of FecB mutation ewes, and these different expression proteions of FecB mutation ewes were all lower than in Non-FecB mutation ewes in mTOR signaling pathway, including ERK1/2, eIF4B and S6 [10]. However, it was not reported that BMPR-1B protein was expressed in vitro, then researched the regulatory pathways of BMPR-1B protein and its interaction molecules (signals) by bioinformatics technology. This research could help to get a deep understanding of sheep prolificacy.

FecB PPIs were identified via the Co-IP/MS method, and the target signal process was screened to better understand the biological pathway of the FecB- interaction protein associated signal transduction functions in ovary extracts of ewes. Sixty-three proteins were successfully identified via *Ovis aries* or *Bos taurus* database matching in Mascot. The most likely associations between these target proteins and BMPR-1B/FecB were computed by the manual thresholding approach and a probabilistic PPI prediction algorithm. Thirty-four high-confidence candidates were identified, 24 of which were at least threefold more abundant between Co-IP of BMPR-1B and FecB groups. TGF-β was identified as the most prominent protein family, including the number of Smads proteins and active proteins. The results identified enrichment for signaling processing via Smads and reproductive processing functions via BMP proteins. In summary, this study provided new insights into PPIs, biological functions, and the roles of the FecB protein.

Proteins are synthesized in ribosomes and transferred to specific organelles to participate in the normal activities of the organism [15]. As a transmembrane protein, BMPR-1B mediates the signal transduction between intracellular and extracellular lumen by participating in life activities and material exchange [16]. GDF5 and BMP4 have been confirmed to be the natural ligands of BMPR-1B, which directly act on secretion of progesterone in ewe granule cells [17, 18]. BMP2 and GDF9 inhibited the release of cAMP and regulated both the synthesis and secretion of progesterone, estradiol, and androstenedione thus affecting ewe ovarian folliculogenesis and litter size [19, 20]. RhoD indirectly affects ewe ovarian folliculogenesis and litter size by participating in the MAPK signaling pathway and in various cellular processes. HSP10 either directly or indirectly participates in cell proliferation apoptosis, inflammatory immune response, and reproductive processes [21, 22].

As a result of the interaction between pituitary gonadotrophins, autocrine, and paracrine factors of the BMP family, immature oocytes are transformed to fully grown oocytes. This presents the meiotic maturation of an oocyte in the pre-ovulary follicles toward fertilization just before ovulation [23]. BMPs participate in multiple cellular activities such as proliferation, cellular differentiation, migration, organization, and apoptosis [24]. BMPR-1B is the major gene of ewe litter size for many sheep breeds, and regulates ewe ovarian oocytes maturation and ovulation to control litter size by the Smads pathway in interaction with the BMP signaling pathway [25]. The Smads pathway is very important in regulating the maturation and ovulation of ewe ovarian oocytes, and cross interacted with other pathways, such as the MAPK signaling pathway, the Cytokine-cytokine receptor interaction pathway, the TGF-β signaling pathway, the Hippo signaling pathway, and the Canonical Wnt/β-catenin pathway [26]. The results of GO, KEGG, and PPi showed that target proteins were enriched during the developmental process, multicellular organismal process, pigmentation, cellular process, biological regulation, TGF-beta signaling pathway, Hippo signaling pathway, and Cytokine-cytokine receptor interaction.

Therefore, according to recent studies and the results presented here target proteins could be concluded to be mainly regulated by the intracellular signal transduction pathway (Smads pathway and p38-MAPK pathway) and the extracellular signal transduction pathway (Erk-MAP pathway). The initial recruitment of primordial follicles is, in fact, independent of gonadotropin hormones and is likely controlled by various intra-ovarian paracrine/autocrine factors, one of which is BMPR-1B. When paracrine/autocrine factors bind to the BMP receptor, the type II receptor transphosphorylates the type I receptor at an intracellular juxtamembrane site that is rich in glycine and serine residues (the GS domain). Consequently, the type I receptor transphosphorylates the R-Smads signaling proteins Smad 1, 2, 3, 5, and 8. These activated R-Smad proteins bind together in multiple protein complexes with Smad 4 (which is also named the common mediator Smad or Co-Smad) [27, 28]. The R-Smad-Co-Smad complex then moves to the nucleus. Inside the nucleus, these complexes then bind to specific areas of DNA and collaborate with particular transcription factors. Thus, they regulate the activity of target genes, activate transcription of downstream BMP4 and GDF5 genes, and regulate follicular development and ovulation [29–31]. Hence, the major determinant of Smad signaling depends on the specificity to binding to type-I receptors. With the synergism of LH, the phosphorylated HSP 10 can close the gap junction between follicle and oocyte, which results in a decreased cAMP level in the oocyte. At the same time, calcium and calmodulin transfer the GnRH signal to the protein binding site of the tyrosine kinase receptor using the p38-MAPK pathway, which achieves the regulation of animal reproductive traits [32, 33]. The results of Ling showed that HSP 10 was involved in the regulation of ovarian granulosa cell apoptosis, which might affect both the follicular and oocyte maturation. The expression of HSP 10 in stromal cells of PCOS patients was low, anti-apoptosis was weakened, and follicular development was suppressed. Furthermore, HSP 10 was highly expressed in theca cells, and the IGF-1/TGF-β pathway was activated, which caused hyperandrogenemia, and inhibited both follicle maturation and ovulation [34]. As a key molecule of the Erk-MAP kinase pathway, Rho family proteins played a bridging role in the intracellular signal transduction [35]. RhoD participated in the Erk-MAP kinase pathway, where it could activate the pathway to participate in cell differentiation, produce cytokines, and initiate cell apoptosis. When RhoD expression was high, the MAPK pathway was activated, the residue of Tyr and Thr was phosphorylated, the ErK pathway was activated, and the phosphorylation levels of c-fos and c-jun increased. At the same time, the Smad 1 and Smad 5 also showed increased expression; thus, the oocyte differentiation and ovulation were induced [36–38].

## Conclusions

CoIP-MS identified 23 proteins that specifically interacted with BMPR-1B proteins within ewes’ ovary extracts. These proteins were primarily related to developmental processes, multicellular organismal processes, and biological regulation. The signal pathway of target proteins (BMP2, BMP4, GDF5, GDF9, RhoD, and HSP 10) was constructed and bioinformatics prediction showed that BMPR-1B interacts with proteins, thus promoting ovulation in ewe ovaries. In summary, the presented results provide new insight into BMPR-1B PPIs and their biological functions for sheep litter size. Furthermore, this study builds a technical foundation for in vitro expression of sheep BMPR-1B. To further study the biological function and for increased specificity of diagnostic reagents, BMPR-1B should be developed as the major gene of sheep.

## Data Availability

The datasets used or analyzed during the current study are available from the corresponding author on reasonable request.

## Abbreviations

BMPR-1B: Bone morphogenetic protein receptors 1B
Co-IP: Co-immunoprecipitation
PPIs: Protein-protein interactions
MS: Mass spectrometry
Erk: Eukaryotic
MAPK: Mitogen-activated protein kinase
FSH: Follicle-stimulating hormone
LH: Luteinizing hormone
PCR–RFLP: Cleaved amplification polymorphism sequence-tagged sites
SDS-PAGE: Sodium dodecyl sulfate- polyacrylamide gel electrophoresis
CPE: Cytopathic effect
GO: Gene ontology
KEGG: Kyoto Encyclopedia of Genes and Genomes
MALDI-TOF/TOF: Matrix assisted laser desorption ionization time of flight mass spectrometry
LC-MS: Liquid chromatograph mass spectrometer
